# Effect of Acute Inspiratory Muscle Exercise on Blood Flow of Resting and Exercising Limbs and Glucose Levels in Type 2 Diabetes

**DOI:** 10.1371/journal.pone.0121384

**Published:** 2015-03-24

**Authors:** Ana Paula dos Santos Corrêa, Cristiano Fetter Antunes, Franciele Ramos Figueira, Marina Axmann de Castro, Jorge Pinto Ribeiro, Beatriz D’Agord Schaan

**Affiliations:** 1 Exercise Pathophysiology Research Laboratory, Hospital de Clínicas de Porto Alegre, Porto Alegre, Rio Grande do Sul, Brasil; 2 Faculty of Health Sciences, University of Sydney, Lidcombe, NSW, Australia; 3 Postgraduate Program in Endocrinology and Department of Internal Medicine, Universidade Federal do Rio Grande do Sul, Porto Alegre, Rio Grande do Sul, Brasil; 4 Postgraduate Program in Pneumology Science, Universidade Federal do Rio Grande do Sul, Porto Alegre, Rio Grande do Sul, Brasil; 5 Postgraduate Program in Cardiology, Universidade Federal do Rio Grande do Sul, Porto Alegre, Rio Grande do Sul, Brasil; Emory University, UNITED STATES

## Abstract

To evaluate the effects of inspiratory loading on blood flow of resting and exercising limbs in patients with diabetic autonomic neuropathy. Ten diabetic patients without cardiovascular autonomic neuropathy (DM), 10 patients with cardiovascular autonomic neuropathy (DM-CAN) and 10 healthy controls (C) were randomly assigned to inspiratory muscle load of 60% or 2% of maximal inspiratory pressure (PImax) for approximately 5 min, while resting calf blood flow (CBF) and exercising forearm blood flow (FBF) were measured. Reactive hyperemia was also evaluated. From the 20 diabetic patients initially allocated, 6 wore a continuous glucose monitoring system to evaluate the glucose levels during these two sessions (2%, placebo or 60%, inspiratory muscle metaboreflex). Mean age was 58 ± 8 years, and mean HbA1c, 7.8% (62 mmol/mol) (DM and DM-CAN). A PImax of 60% caused reduction of CBF in DM-CAN and DM (P<0.001), but not in C, whereas calf vascular resistance (CVR) increased in DM-CAN and DM (P<0.001), but not in C. The increase in FBF during forearm exercise was blunted during 60% of PImax in DM-CAN and DM, and augmented in C (P<0.001). Glucose levels decreased by 40 ± 18.8% (P<0.001) at 60%, but not at 2%, of PImax. A negative correlation was observed between reactive hyperemia and changes in CVR (Beta coefficient = -0.44, P = 0.034). Inspiratory muscle loading caused an exacerbation of the inspiratory muscle metaboreflex in patients with diabetes, regardless of the presence of neuropathy, but influenced by endothelial dysfunction. High-intensity exercise that recruits the diaphragm can abruptly reduce glucose levels.

## Introduction

Cardiovascular autonomic neuropathy causes increased morbidity and mortality to diabetic patients [1]. Exercise intolerance is one of its major clinical manifestations, which translates into low levels of physical activity and poor cardiorespiratory fitness; this, in turn, is an independent predictor of all-cause mortality [2]. The limited exercise response experienced by patients with diabetic autonomic neuropathy may result from reduced responses in heart rate, blood pressure, and cardiac output [3], as well as decreased strength and endurance of inspiratory muscles [4, 5] and impaired endothelium-dependent vasodilatory responses [6].

Respiratory muscle weakness in diabetic patients may be associated with peripheral [7] and autonomic neuropathy [4,5]. Another possible explanation may be the activation of the inspiratory muscle metaboreflex, which occurs when fatiguing contractions of the inspiratory muscles during exercise cause accumulation of metabolic products. These products stimulate skeletal muscle neural afferents, resulting in increased sympathetic vasoconstrictor activity [8], thereby decreasing the locomotor muscle perfusion. Thus, the respiratory muscles "steal" blood flow away from the locomotor muscles [9]. Although physiologic, the inspiratory muscle metaboreflex may affect patients’ symptoms and the ventilatory response to exercise [10]. Blunting of the inspiratory muscle metaboreflex with improvement in limb blood flow and endurance capacity can be obtained by endurance training in healthy individuals [11] and by respiratory muscle training in patients with heart failure [12,13]. Accordingly, improved strength of the inspiratory muscles can also be obtained by inspiratory muscle training in diabetic patients [4]. However, it is not yet known if this kind of training activates or blunt the inspiratory muscle metaboreflex in patients with diabetes.

As muscle metaboreflex activity and brachial artery flow-mediated dilatation are both impaired in hypertensive and diabetic patients [14], endothelial dysfunction could partially explain the impaired leg blood flow regulation response to exercise, further limiting exercise capacity in diabetic subjects [15]. It is possible that activation of the inspiratory muscle metaboreflex would cause excessive reduction in blood flow control during exercise in the presence of diabetic autonomic neuropathy, as these patients would experience greater impairment of endothelium-dependent vasodilation [14], but this has yet to be investigated. We hypothesized that high inspiratory muscle loading would determine different blood flow of resting and exercising limbs in patients with diabetes as compared to healthy subjects, as well as that cardiovascular autonomic neuropathy would cause further derangements in these responses.

The present study was conducted to evaluate the effects of inspiratory loading on blood flow of resting and exercising limbs in patients with diabetic autonomic neuropathy, comparing their responses to those elicited by the same protocol in controls and diabetic patients without autonomic neuropathy.

## Materials and Methods

### Patients and design

The protocol was approved by the Hospital de Clínicas de Porto Alegre (HCPA) Research Ethics Committee. All subjects provided written informed consent for participation. Ten patients with type 2 diabetes without autonomic neuropathy (DM) were matched to 10 patients with type 2 diabetes with moderate and severe cardiovascular autonomic neuropathy (DM-CAN). The presence or absence of autonomic neuropathy was assured using the five noninvasive cardiovascular reflex tests proposed by Ewing, as previously described [20, 21]. They were recruited from the ambulatory of the HCPA, Brazil. Exclusion criteria were body mass index (BMI) ≥ 30 kg.m^2^, exercise-induced asthma, infectious, osteoarticular, cardiac or pulmonary diseases, as well as regular alcohol or tobacco consumption in the preceding 6 months. A group of 10 healthy controls (C) were selected to match the DM and DM-CAN groups in age, gender, and BMI.

### Protocol

Subjects came to the laboratory on three separate days for completion of the study protocols. On the first day, pulmonary function, inspiratory muscle function and cardiovascular autonomic function were evaluated. On the second day, individuals underwent endothelial function assessment and cardiopulmonary exercise testing. On the third day, at least two days after the above-described evaluations, subjects underwent a protocol designed to elicit the inspiratory muscle metaboreflex, so as to evaluate its impact on resting hemodynamic parameters, maximal inspiratory muscle pressure, calf blood flow (CBF) and forearm blood flow (FBF). All experiments were performed in a temperature-controlled room and all subjects were in the fasting state (at least for 3 hours), had refrained from consuming caffeinated beverages and alcohol for at least 12 hours, and had not exercised for at least 48 hours prior to the evaluation.

#### Pulmonary function

Measurements of forced vital capacity (FVC), forced expiratory volume in 1 second (FEV1), and maximal voluntary ventilation (MVV) were obtained with a computerized spirometer (Eric Jaeger, GmbH, Würzburg, Germany), as recommended by the American Thoracic Society [16], and results were expressed as percentage of predicted value [17].

#### Inspiratory muscle function

Inspiratory muscle function testing was performed using a pressure transducer (MVD-500 V.1.1 Microhard System; Globalmed, Porto Alegre, Brazil), connected to a system with two unidirectional valves (DHD Inspiratory Muscle Trainer, Chicago, IL) as previously described [12]. In short, the maximal inspiratory mouth pressure (PImax) was determined during deep inspiration from residual volume against an occluded airway with a small air leak. The test was repeated several times to obtain 6 measurements with less than 10% variation [18]. Predicted values were corrected for age and gender [19]. Inspiratory muscle endurance (Pthmax) was determined by an incremental test and expressed as a percentage of PImax (Pthmax/PImax).

#### Cardiovascular autonomic function testing

The presence of cardiovascular autonomic neuropathy was verified using the five noninvasive cardiovascular reflex tests proposed by Ewing, as previously described [20] and standardized in our institution [21]. Three tests were used to evaluate the heart rate response to *1*) deep breathing, *2*) lying-to-standing heart rate ratio, and *3*) the Valsalva maneuver. Two tests of blood pressure control were also calculated during *1*) orthostatic hypotension and *2*) sustained handgrip. All tests were performed three times, and the mean value was used for analysis. Two or more abnormal tests were deemed diagnostic of cardiovascular autonomic neuropathy [20, 21]. Heart rate (HR) variation was assessed while patients were asked to breathe deeply at a rate of 6 breaths per minute while being monitored on 3-lead electrocardiography. The maximum and minimum HR during each breathing cycle were measured, and the mean difference of 6 cycles was calculated. Heart rate ratio test was determined after 10 min of rest in the supine position, and HR variability was determined by calculating the maximal to minimal HR ratio: the longest R-R interval, measured around the 30^th^ beat after standing up, to the shortest R-R interval, measured around the 15^th^ beat after standing up. The Valsalva test consisted of forced exhalation into a mouthpiece with a pressure of 40 mmHg for 15 s, and the ratio of the maximum R-R after the maneuver to the minimum R-R during the maneuver was calculated. Orthostatic hypotension was defined as a decrease in systolic blood pressure of 30 mmHg when the individual voluntarily changed from the supine to the standing position. Measurements were obtained every minute for at least 3 min. Sustained muscle contraction as measured by handgrip dynamometer. The dynamometer was first squeezed to isometric maximum; then, patients were asked to hold at 30% maximum for 5 min. A rise in diastolic blood pressure of < 10mmHg is considered an abnormal response.

#### Endothelial function

Reactive hyperemia were measured using cuffs placed at the upper and lower arm. In the lower arm, a rapid cuff inflator was used to occlude venous outflow (50–60 mmHg), and three blood flow recordings were obtained each minute for 3 min. Thereafter, the upper-arm cuff, which induces hyperemia, was occluded at 250 mmHg for 5 min, with pressure released in 10 s intervals over 1 min. Reactive hyperemia was calculated using the percentage of dilatation after the post-occlusion flow 5 min in relation to baseline [22].

#### Cardiopulmonary exercise testing

The maximal incremental exercise test was performed on an electrically braked cycle ergometer (ER-900, Ergoline, Jaeger, Würzburg, Germany) with minute increments of 10 W for DM-CAN, 15 W for DM, and 20 W for C. During testing, gas exchange variables were measured by a previously validated system (Oxycon Delta, VIASYS, Healthcare GmbH, Jaeger, Germany).

#### Inspiratory muscle exercise session protocol (2% and 60% of PImax)

Induction of the inspiratory muscle metaboreflex was performed according to apreviously described protocol [23]. In short, subjects breathed continuously through a two-way valve (Hans Rudolph, 2600 series, Shawnee, KS, USA) with low resistance (2% of PImax) connected to an inspiratory resistance obtained by a threshold inspiratory muscle trainer (DHD inspiratory muscle trainer, Chicago, IL) or to a POWERbreathe inspiratory muscle trainer (Southam, UK) for higher inspiratory pressures (60% of PImax). The sequence of performing the experiments, induction of the inspiratory muscle metaboreflex (60% of PImax) or allocation to the placebo experiment (2% of PImax) was randomly assigned, and the experiments were separated by a 40 min interval [11, 23] Baseline data were collected during 5 minutes of spontaneous breathing in both protocols. After this period, controlled breathing for 3 min was initiated for both protocols, and individuals maintained a breathing frequency (*f*b) of 15 breaths per min and a duty cycle (T_I_/T_Tot_) of 0.7 by listening to an audio signal with distinct inspiratory and expiratory tones in both protocols. Inspiratory pressure was continuously recorded and displayed on a computer screen and a 10-point Borg scale [24] was used to access inspiratory effort at task failure. The session took as long as patients would support until diaphragm muscles to fatigue (approximately 5 min). Inability to maintain breathing was defined as a reduction of PImax to less than 80% of the prescription during 3 consecutive breaths. For the placebo experiments (2% of PImax) a controlled breathing for 3 min was also conducted and the measures during the 2% loading were interrupted at 5 min. The CBF responses were assessed as subjects breathed under high (60% of PImax) and low (2% of PImax) inspiratory pressures.

#### Forearm exercise experiment after respiratory exercise at 2% and 60% of PImax

Immediately after induction of the inspiratory muscle metaboreflex and the placebo experiment, subjects began the forearm exercise, which consisted of repetitive maximal voluntary contractions of the dominant forearm on a hand dynamometer (Kratos, DLC, Cotia, Brazil) that was measured at baseline, maintained for 10 s and released for 30 s until task failure. The session took as long as patients would support until forearm fatigue (approximately 7 min). During the relaxation phase, FBF was measured by venous occlusion plethysmography (Hokanson, TL-400, Bellevue, USA) and all patients were encouraged verbally throughout the isometric contraction in order to maintain handgrip force at target.

#### Ventilatory and hemodynamic measures

During induction of the inspiratory muscle metaboreflex and the placebo experiment, ventilatory and hemodynamic variables were measured as previously described [23]. In short, *fb*, arterial oxygen saturation by pulse oximetry (Sp_O2_), and end-tidal carbon dioxide (ETCO_2_) were measured by oxycapnography (Takaoka Oxicap, São Paulo, Brazil). Mean arterial blood pressure (MAP) was measured on the non-dominant arm with an automated sphygmomanometer at each minute of the test (Dinamap 1846 SX/P, Critikon, Tampa, USA). Calf blood flow and FBF were measured by venous occlusion plethysmography (Hokanson, TL-400, Bellevue, USA). Calf vascular resistance (CVR) and forearm vascular resistance (FVR) ware calculated as CBF/MAP and FBF/MAP, respectively [25]. End-tidal carbon dioxide was maintained at eupneic levels during both protocols via addition of CO_2_ into the inspiratory circuit.

#### Metabolic evaluation by the continuous subcutaneous glucose monitoring system (CGMS)

One subgroup of 6 patients with diabetes was randomly assigned to two sessions of inspiratory muscle exercise either with 2% of PImax (placebo condition) or 60% of PImax (metaboreflex activation), in different days with one week apart. Subjects were admitted to the laboratory in the morning, when the CGMS device was placed (Medtronic Mini-Med, Northridge, CA). The sensor was inserted through a needle into the subcutaneous tissue of the abdominal wall using a spring-loaded device (Senserted Medtronic, Northridge, USA) 24h before the beginning of the protocols and maintained through the exercise sessions until a 30 min of recovery after the sessions was reached, when it was removed. Glucose measurements were obtained every 10 s and averaged every 5 min, for a total of 288 readings per day. Sensor readings were calibrated with a glucose monitor (Accu-Check Performa, Roche Diagnostics, Mannheim, Germany) using 4 finger stick blood samples obtained over the 24-h period. Patients were previously instructed about the operation of the monitor. Subjects were asked to closely match their daily nutritional intake.

### Statistical Analysis

A sample size of 10 individuals per group was estimated to detect a 10% difference in CBF response, with for a statistical power of 0.8 and an alpha of 0.05. Values are reported as mean ± SD. All analyses were performed using the Statistical Package for the Social Sciences 18.0 for Windows software (SPSS Inc., Chicago, IL). Generalized estimating equations (GEE) were used to compare differences in patients’ characteristics and baseline values among groups. The GEE model for repeated measures was used to compare respiratory and hemodynamic measurements during the inspiratory muscle metaboreflex protocol, followed by Bonferroni’s *post-hoc* test for multiple comparisons. Pearson’s correlation coefficient and multiple linear regression analyses were performed to determine the relationship between VO2 peak, delta of CVR and baseline HbA1c and PImax variables after the protocols. Categorical variables were compared by means of Fisher’s exact test. Findings were considered significant at p < 0.05.

## Results

### Baseline characteristics

The baseline characteristics, resting hemodynamics, pulmonary/ inspiratory muscle function and cardiopulmonary exercise testing results of the subjects are shown in Table 1. Groups were similar in age, weight, and BMI. As expected, plasma glucose and glycated hemoglobin (HbA1c) levels were higher in the DM and DM-CAN groups as compared to C. The reactive hyperemia in the study groups was decreased in both DM and DM-CAN subjects as compared with the C group. Resting HR was higher in DM-CAN as compared to the other groups. Lower MVV, PImax, endurance time, R_peak_ and V̇O_2peak_ were observed in DM and DM-CAN as compared to C. Lower Pthmax/PImax, and HR_peak_ were observed in DM-CAN as compared to C. The FEV_1_ was lower in DM-CAN as compared with the C and DM groups. The FVC was lower in DM and DM-CAN as compared to C, and even lower in the DM-CAN as compared with the DM group.

**Table 1:** Clinical characteristics, resting hemodynamics, pulmonary/inspiratory muscle function and cardiopulmonary exercise testing of the three study groups.

	C (n = 10)	DM (n = 10)	DM-CAN (n = 10)
**General**			
Male: female ratio	6/4	4/6	4/6
Age, y	56 ± 7	59 ± 8	59 ± 9
Body mass index, kg.m^-2^	25 ± 3	26 ± 7	27 ± 1
Diabetes duration, y	-	8 (6–12)	11 (6–15)
HbA1c %	5 (5–6)	7 (7–9)*	8 (8–9)* ^†^
Fasting plasma glucose,mg.dL^-1^	89 ± 6	144 ± 21*	141 ± 26*
Total cholesterol, mg.dL^-1^	165 ± 26	190 ± 23	186 ± 30
HDL cholesterol, mg.dL^-1^	42 ± 9	45 ± 8	45 ± 8
GFR, ml/min/1.73m^2^	92.8 ± 20.8	79.2 ± 20.3	72.8 ± 14.5
**Resting hemodynamics**			
MAP, mmHg	93 ± 8	91 ± 7	95 ± 7
HR, beats/min	68 ± 6	74 ± 11	80 ± 7*
CBF, ml/min.100 ml	3.6 ± 1	3.5 ± 0	2.7 ± 0
CVR, units	27 ± 7	28 ± 7	33 ± 4
Handgrip force, N	39 ± 13	32 ± 5	34 ± 12
Flow mediated dilatation, %	12 ± 2	6 ± 2*	4 ± 3*
**Medications, n (%)**			
Metformin	-	8 (80)	9 (90)
Sulfonylureas	-	2 (20)	1 (10)
ACE inhibitor	-	1 (10)	3 (30)
Diuretics	-	4 (40)	2 (20)
Statins	-	1 (10)	1 (10)
Beta blockers	-	1 (10)	1 (10)
**Pulmonary function**			
FEV1, % predicted	92 ± 2	88 ± 4	81 ± 5* ^†^
FVC, % predicted	99 ± 1	99 ± 2	94 ± 4* ^†^
MVV, % predicted	116 ± 13	80 ± 1*	79 ± 1*
**Inspiratory muscle function**			
PI_max_, cmH_2_O	111 ± 13	76 ± 17*	80 ± 16*
PI_max_, % predicted	109 ± 10	86 ± 10*	84 ± 9*
Pthmax/PImax, %	73 ± 14	70 ± 20	50 ± 17*
Endurance time, s	538 ± 129	302 ± 58*	238 ± 84*
**Cardiopulmonary exercise test**			
HR _peak_, beats/min	168 ± 13	159 ± 12	145 ± 17*
V̇O_2peak_, mL.Kg^-1^.min^-1^	37 ± 6	25 ± 3*	20 ± 1*
R_peak_	1.2 ± 0	1.1 ± 0*	1.1 ± 0*

Values expressed as mean ± SD or n (%). C, control, DM, diabetes mellitus; DM-CAN, diabetes mellitus and cardiovascular autonomic neuropathy; GFR, glomerular filtration rate; MAP, mean arterial pressure; CBF, calf blood flow; CVR, calf vascular resistance. HR, heart rate; ACE, angiotensin-converting enzyme; HbA1c, glycated hemoglobin; FEV_1_, forced expiratory volume in 1 s; FVC, forced vital capacity; MVV, maximal voluntary ventilation; PImax, maximal inspiratory pressure; Pthmax, inspiratory endurance determined during incremental test; V̇O_2_peak, peak oxygen uptake; R, respiratory exchange ratio. Analysis: Generalized estimating equations (GEE) for repeated measures.

* P<0.05 *vs*. C;

^†^ P<0.05 *vs*. DM.

### Inspiratory muscle exercise session protocol (2% and 60% of PImax)

**Fig 1:**
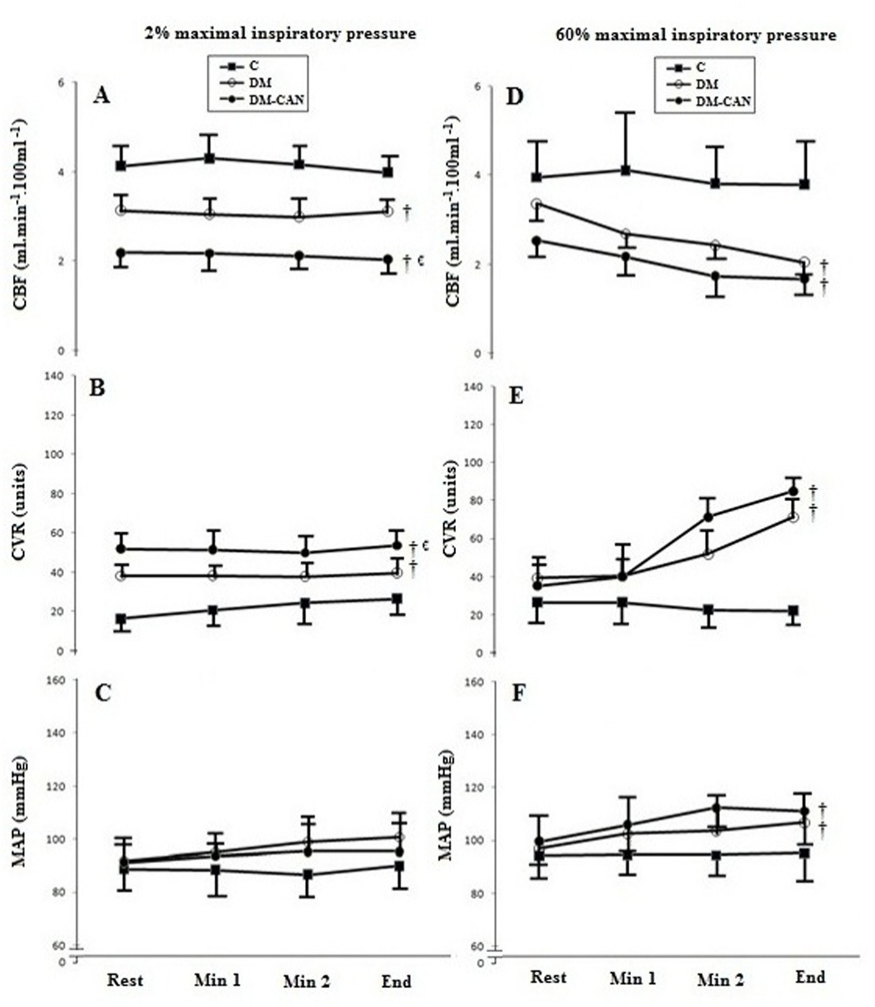
Calf blood flow (CBF), calf vascular resistance (CVR), and mean arterial pressure (MAP) at baseline, minute 1, minute 2 and at the end of the placebo experiment (2% PImax, left panels) and during induction of the inspiratory muscle metaboreflex (60% PImax., right panels). Groups: Controls (C, closed squares), patients with type 2 diabetes without autonomic neuropathy (DM, open circles), and patients with type 2 diabetes and cardiovascular autonomic neuropathy (DM-CAN, closed circles). Data expressed as mean ± SD. Analysis: Generalized estimating equations (GEE) for repeated measures, followed by Bonferroni’s post hoc test. Panel A: group (p<0.001), time (p = 0.72) and interaction (p<0.001); Panel B: group (p = 0.03), time (p = 0.34) and interaction (p = 0.001); Panel C: group (p = 0.84), time (p = 0.22) and interaction (p = 0.15); Panel D: group (p<0.001), time (p = 0.002) and interaction (p<0.001); Panel E: group (p<0.001), time (p<0.001) and interaction (p<0.001); Panel F: group (p<0.001), time (p<0.001) and interaction (p<0.001); ^†^p<0.05 *vs*. C; ^€^p<0.05 *vs*. DM.

These data are shown in Table 2 and Fig. 1. Patients from the DM-CAN group did not maintain breathing during inspiratory effort (128 ± 29 s) as long as the others, quitting earlier than both DM (207 ± 30 s) and C (326 ± 44 s) (P < 0.01). All subjects were able to maintain a breathing frequency of ~15 breaths/min and a prolonged T_I_/T_Tot_ of ~0.7 for fatiguing and control protocols. The HR was lower at the end point of the 60% protocol in DM-CAN when compared with C and DM. The ETCO_2_ remained at eupneic levels during both protocols. Fig. 1 shows the reduction in CBF and increment in CVR and MAP in the DM and DM-CAN groups as compared with C as the inspiratory muscle metaboreflex was induced.

**Table 2:** Resting calf experiment during respiratory exercise and forearm exercise experiment after respiratory exercise at 2% and 60% of PImax in the subjects studied.

Resting Calf					
		Baseline	1 min	2 min	End
**2% PImax; TI/TTot, 0.7**					
Time, s					
	C	-	-	-	300 ± 0
	DM	-	-	-	300 ± 0
	DM-CAN	-	-	-	300 ± 0
HR (beats.min^-1^)					
	C	83 ± 3	82 ± 8	83 ± 10	82 ± 9
	DM	72 ± 11	74 ± 10	75 ± 12	75 ± 12
	DM-CAN	75 ± 15	74 ± 15	75 ± 16	75 ± 12
*f*_b_ (breaths/min^-1^)					
	C	15 ± 0	16 ± 1	16 ± 1	15 ± 1
	DM	16 ± 1	15 ± 0	15 ± 1	15 ± 0
	DM-CAN	15 ± 0	16 ± 1	16 ± 1	15 ± 1
ETCO_2_ (mmHg)					
	C	29 ± 3	31 ± 1	30 ± 0	30 ± 1
	DM	24 ± 3	23 ± 4	23 ± 4	23 ± 3
	DM- CAN	24 ± 3	22 ± 2	23 ± 2	22 ± 3
SpO_2_ (%)					
	C	98 ± 1	97 ± 1	98 ± 1	98 ± 0
	DM	98 ± 0	97 ± 1	97 ± 1	97 ± 1
	DM-CAN	98 ± 0	98 ± 0	98 ± 0	97 ± 1
**60% PImax; TI/TTot, 0.7**					
Time, s					
	C	**-**	**-**	**-**	326 ± 44
	DM	**-**	**-**	**-**	207 ± 30
	DM-CAN	**-**	**-**	**-**	128 ± 29
HR (beats.min^-1^)					
	C	71 ± 9	74 ± 8	80 ± 9	81 ± 4
	DM	80 ± 13	86 ± 8	94 ± 4	81 ± 13
	DM-CAN	82 ± 2 ^¥^	78 ± 7	98 ± 1	76 ± 8* ^†^ ^€^ ^¥^
f_b_ (breaths/min^-1^)					
	C	15 ± 0	15 ± 0	15 ± 1	15 ± 0
	DM	15 ± 1	16 ± 1	16 ± 1	15 ± 1
	DM-CAN	16 ± 0	18 ± 1	17 ± 0	16 ± 1
ETCO_2_ (mmHg)					
	C	32 ± 2	33 ± 0	31 ± 0	33 ± 2
	DM	23 ± 4	22 ± 3	24 ± 3	22 ± 3
	DM-CAN	24 ± 2 ^¥^	26 ± 1	24 ± 0	25 ± 0
SpO_2_ (%)					
	C	98 ± 0	98 ± 0	98 ± 0	97 ± 1
	DM	98 ± 0	98 ± 0	98 ± 1	97 ± 1
	DM-CAN	98 ± 0	98 ± 1	98 ± 1	97 ± 1
**Forearm exercise**					
		**End-Inspiratory Load**	**1 min**	**2 min**	**End**
**2% PImax; TI/TTOT, 0.7; *f***_**b**_ **, 15**					
Time, s					
	C	-	-	-	300 ± 0
	DM	-	-	-	300 ± 0
	DM-CAN	-	-	-	300 ± 0
HR (beats.min^-1^					
	C	71 ± 8	71 ± 8	73 ± 9	75 ± 7
	DM	72 ± 8	71 ± 10	68 ± 12	77 ± 12
	DM-CAN	77 ± 7	77 ± 8	75 ± 8	79 ± 8
SpO_2_ (%)					
	C	98 ± 0	98 ± 0	98 ± 0	98 ± 0
	DM	98 ± 0	97 ± 1	97 ± 1	97 ± 2
	DM-CAN	98 ± 0	96 ± 2	95 ± 4	97 ± 1
**60% PImax; TI/TTOT, 0.7;*f***_**b**_ **, 15**					
Time, s					
	C	-	-	-	326 ± 50
	DM	-	-	-	209 ± 31
	DM-CAN	-	-	-	159 ± 34
HR (beats.min^-1^)					
	C	76 ± 14	76 ± 13	80 ± 17	81 ± 12
	DM	73 ± 10	70 ± 7	72 ± 15	76 ± 15
	DM-CAN	83 ± 3	77 ± 7	78 ± 6	76 ± 8
SpO2 (%)					
	C	97 ± 1	97 ± 1	98 ± 0	98 ± 1
	DM	97 ± 1	97 ± 1	97 ± 1	96 ± 4
	DM-CAN	96 ± 3	95 ± 4	93 ± 8	95 ± 3

Values expressed as means ± SD. C, control, DM, diabetes mellitus; DM-CAN, diabetes mellitus and cardiovascular autonomic neuropathy; HR, heart rate; *f*b, breathing frequency; SpO_2_, oxygen saturation; ETCO_2_, end-tidal carbon dioxide; TI/TTot, duty cycle. Analysis: Generalized estimating equations (GEE) for repeated measures.

* P<0.05 *vs*. C;

^†^ P<0.05 *vs*. DM;

^€^ P<0.05 different from baseline;

^¥^ different from protocol at 2% PImax.

#### Forearm blood flow increment is blunted after inspiratory loading in diabetes

**Fig 2:**
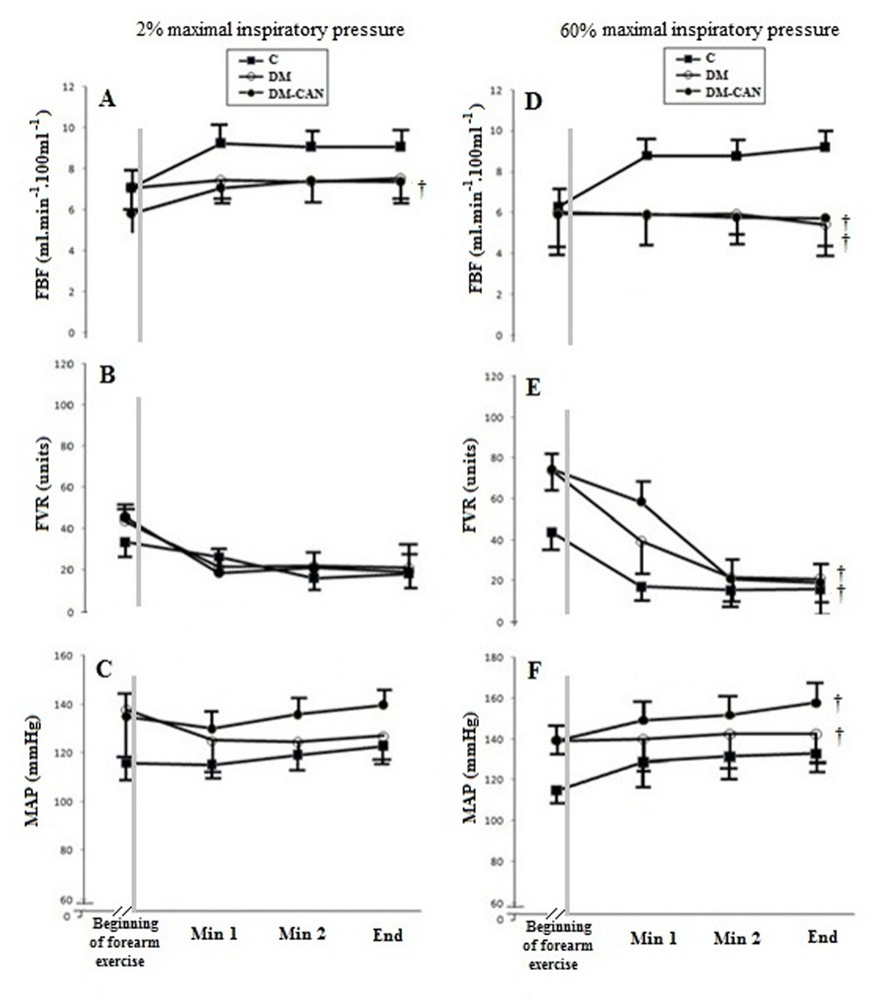
Forearm blood flow (FBF), forearm vascular resistance (FVR), and mean arterial pressure (MAP) immediately after the end of inspiratory loading (onset of forearm exercise), at minute 1, minute 2 and at the end of forearm exercise. Groups: Controls (C, closed squares), patients with type 2 diabetes without autonomic neuropathy (DM, open circles), and patients with type 2 diabetes and cardiovascular autonomic neuropathy (DM-CAN, closed circles). Data expressed as mean ± SD. Analysis: Generalized estimating equations (GEE) for repeated measures, followed by Bonferroni’s post hoc test. Panel A: group (p<0.05), time (p<0.05), interaction (p<0.05); Panel B: group (p = 0.29), time (p = 0.49), interaction (p = 0.34); Panel C: group (p = 0.34), time (p = 0.74), interaction (p = 0.16); Panel D: group (p<0.001), time (p<0.001), interaction (p<0.001); Panel E: group (p<0.001), time (p<0.001), interaction (p<0.001); Panel F: group (p<0.001), time (p<0.001), interaction (p<0.001); ^†^p<0.05 *vs*. C.

These data are shown in Fig. 2 and Table 2. Time to fatigue during forearm exercise was reduced in the 60% protocol in the DM (209 ± 31 s) and DM-CAN (159 ± 34 s) groups as compared with C (326 ± 50 s), *P*< 0.001. The intermittent static handgrip exercise after inspiratory muscle loading at 60% of PImax resulted in similar SpO_2_ and HR responses among the groups studied. Fig. 2 shows the blunted increment in FBF during forearm exercise for the induction period of the inspiratory muscle metaboreflex in DM and DM-CAN as compared with C. Conversely, forearm vascular resistance (FVR) and MAP during exercise were higher in DM and DM-CAN than in C.

Calf vascular resistance was negatively associated with baseline values of Pimax (r = -0.492; P = 0.006), reactive hyperemia (r = - 0.746, P<0.001) and VO2peak (r = -0.680; P<0.001) on pooled analysis of all groups. On multiple linear regression, only reactive hyperemia remained significantly correlated with changes in delta CVR (Beta coefficient = -0.44, P < 0.034).

#### Metabolic evaluation (CGMS) showing lowering of glucose levels during the metaboreflex induction in diabetes

**Fig 3:**
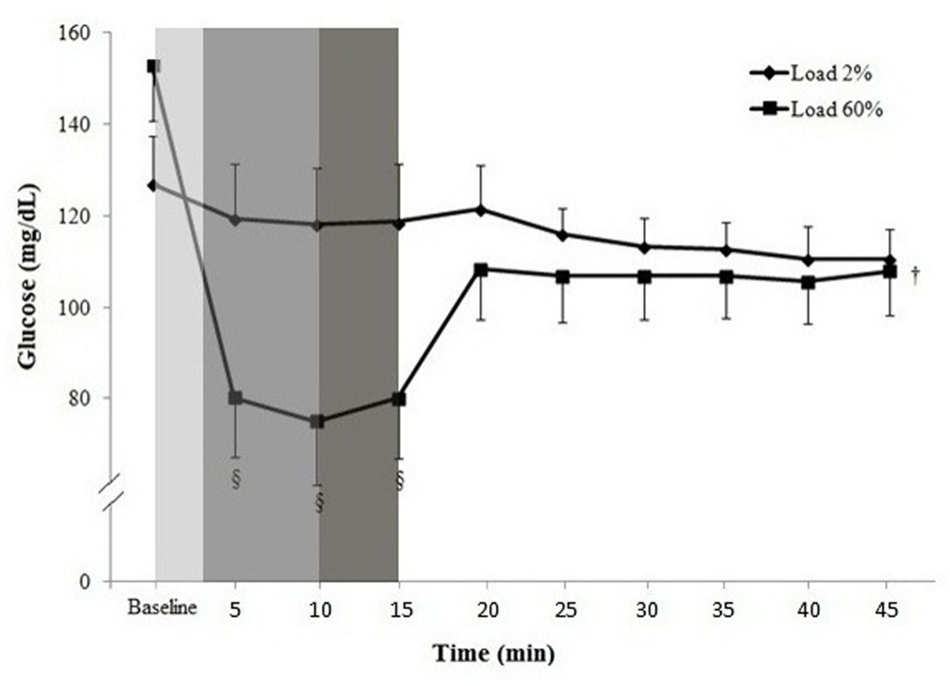
Glucose levels as measured by the continuous subcutaneous glucose monitoring system (CGMS) at rest, breath control (grey bar), load (dark bar), handgrip exercise (black bar) and recovery period during placebo experiment (2% PImax) and during induction of the inspiratory muscle metaboreflex (60% PImax) in a random subgroup of 6 diabetic patients, both without cardiovascular autonomic neuropathy and with cardiovascular autonomic neuropathy. Data expressed as mean ± SD. Analysis: Generalized estimating equations (GEE) for repeated measures, followed by Bonferroni’s post hoc test. Group (Pimax load 2% *vs*. Pimax load 60%, p<0.05), time (p<0.001) and interaction (p<0.05). §p<0.05 *vs*. baseline; ^†^p<0.05 *vs*. PImax load 2%.

Fig. 3 shows glucose levels obtained during the metaboreflex induction in a random subgroup of six diabetic patients, four with DM-CAN and two with DM. At the induction of the inspiratory muscle metaboreflex (60% PImax), glucose levels decreased significantly and remained low until the end of the test (15 min, P<0.001). This was not seen during the placebo experiment, when a minimal PImax (2% PImax) was applied. Glucose levels remained low during the recovery period (30 min, P<0.001).

## Discussion

This study shows that voluntary efforts against an inspiratory resistive load of 60% of PImax cause exaggerated vasoconstriction of the resting calf in patients with type 2 diabetes, suggesting an exacerbation of the inspiratory muscle metaboreflex. These fatiguing levels of inspiratory muscle force translated into reduced circulatory responses to forearm exercise. These derangements were determined by the diabetic state *per se*, not by autonomic neuropathy, as no differences were observed between patients with or without this complication. In addition, changes in the peripheral vasoconstrictor response to inspiratory metaboreflex activation were inversely related to reactive hyperemia. This study also shows that glucose levels decreased significantly during the inspiratory muscle metaboreflex activity, and remained low for at least 30 minutes after cessation of exertion.

Several studies have shown that the inspiratory metaboreflex generated by fatiguing inspiratory muscle work elicits a sympathetically mediated vasoconstrictor response in the resting limbs of healthy subjects [8, 26]. The prevailing level of inspiratory muscle work is inversely related to the time to the limit of tolerance during very heavy cycling exercise [8,10] and to the magnitude of exercise-induced limb muscle fatigue [26,27]. It has also been suggested that inspiratory muscle work can alter exercise tolerance, a phenomenon probably mediated by its influence on limb perfusion. In healthy subjects, inspiratory metaboreflex activation may be particularly important during sustained heavy-intensity exercise, as it modulates the competition for blood flow between the respiratory and locomotor muscles [27]. In agreement with these findings, inspiratory muscle fatigue increased forearm vascular resistance immediately at the onset of exercise and the time to fatigue during handgrip exercise in the C group. However, the forearm vascular resistance was blunted in patients with diabetes after inspiratory loading when compared with controls. These findings suggest that inspiratory muscle metaboreflex activation restricted forearm hyperemic responses to handgrip exercise in these patients, possibly by an already established reduction in sympathetic and vagal function [28]. Interestingly, these findings in patients with diabetes are similar to those reported previously in chronic heart failure, where excessive inspiratory muscle work contributes to augmented respiratory muscle metaboreflex stimulation, derangements that are associated with exercise limitation [23].

It is known that autonomic neuropathy can impair hemodynamic responses to cardiovascular stress, and also cause decreased heart rate variability [1] and damage to the autonomic nerve fibers that innervate the heart and blood vessels [29]. These abnormalities in heart rate control and vascular dynamics may be involved in changes in leg blood flow caused by limb vasoconstriction, leading to an impaired inspiratory muscle metaboreflex. However, although a clear rationale implicates autonomic neuropathy as a possible determinant of exacerbated metaboreflex in diabetic patients, this was not demonstrated in the present study. Although our patients with autonomic dysfunction exhibited greater derangements in pulmonary function as compared to those without neuropathy, exercise tolerance was low in both diabetes groups, regardless of the presence of neuropathy. These results suggest diabetic-specific causes of exercise intolerance rather than neuropathy. These could be a combination of pulmonary derangements [4,5] and impaired endothelium-dependent vasodilatory responses [6].

In healthy subjects metaboreflex activation results in greater increases of sympathetic outflow during heavy exercise, resulting in attenuated reactive hyperemia, an indicator of endothelial function. In type 2 diabetes it is possible that the increased sympathetically-mediated vasoconstriction (metaboreflex activation) resulted in augmented vasoconstriction to a point that the already dysfunctional endothelium [30] was unable to allow for increased vasodilation. This is in accordance with previous data showing that subjects with diabetes had attenuated sensitivity to vasodilatory agents when leg blood flow was evaluated during the infusion of ATP; impaired peripheral vasodilatation may affect their ability to tolerate higher intensities of exercise due to low leg blood flow [31]. In contrast, in patients with diabetes and no endothelial dysfunction, functional sympatholysis was intact during moderate exercise [32].

Leg blood flow is reduced in type 2 diabetic subjects during exercise, a response that is correlated with endothelial function [15]. This impaired leg blood flow response to exercise may be involved in the limited exercise capacity in diabetes, as this was shown to be associated with longer duration of diabetes and the microvascular [33] and macrovascular complications of the disease [15]. Another potential mechanism involved in the leg blood flow response to exercise would be metabolic control, as acute hyperglycemia attenuates endothelium-dependent vasodilation in humans [34]. The significant correlation between calf vascular resistance changes and reactive hyperemia in diabetes may represent a dysfunction in the control of blood flow. These data suggest that exacerbation of the inspiratory muscle metaboreflex is a manifestation of diabetes itself, and that impairment in endothelial function may at least partly account for this phenomenon, contributing to exercise limitation in patients with diabetes.

The abrupt decrease in glucose levels observed during inspiratory loading (~40% reduction at 60% of PImax) was of similar magnitude to the acute reduction in glucose levels observed after acute aerobic exercise in a similar population [35]. It is well known that conventional exercise can acutely reduce glucose levels in diabetic subjects [35,36], as it results in acute increases in insulin sensitivity and high muscle glucose uptake [36,37]. However, very intense limb exercise can actually prevent glucose levels from declining if it is intense enough to elicit a rise in counter-regulatory hormones, sometimes even resulting in elevated glucose levels [38]. This is consistent with subsequent findings of increased GLUT4 protein content in the sheep diaphragm induced by chronic inspiratory resistive flow [39].

In conclusion, inspiratory muscle loading of 60% of PImax caused an exacerbation of the inspiratory muscle metaboreflex in subjects with diabetes. This phenomenon occurred regardless of the presence of neuropathy, but was influenced by endothelial dysfunction. High-intensity exercise that recruits the diaphragm can reduce glucose levels abruptly, and this is an avenue that should be explored for its potential relevance to future clinical practice.
